# Object recognition tasks in rats: Does sex matter?

**DOI:** 10.3389/fnbeh.2022.970452

**Published:** 2022-08-12

**Authors:** Marcela Becegato, Regina H. Silva

**Affiliations:** ^1^Behavioral Neuroscience Laboratory, Department of Pharmacology, Federal University of São Paulo, São Paulo, Brazil; ^2^MaternaCiência, Federal University of São Paulo, São Paulo, Brazil

**Keywords:** cognition, behavioral task, spatial memory, ovariectomy, vaginal lavage

## Abstract

Novelty recognition tasks based on object exploration are frequently used for the evaluation of cognitive abilities and investigation of neurobiological and molecular aspects of memory in rodents. This is an interesting approach because variations of the object recognition tasks focus on different aspects of the memory events such as novelty, location, context, and combinations of these elements. Nevertheless, as in most animal neuroscience research, female subjects are underrepresented in object recognition studies. When studies include females, the particularities of this sex are not always considered. For example, appropriate controls for manipulations conducted exclusively in females (such as estrous cycle verification) are not included. In addition, interpretation of data is often based on standardizations conducted with male subjects. Despite that, females are frequently reported as deficient and unable to adequately perform some memory tests. Thus, our study aims to review studies that describe similarities and differences between male and female performances in the different variations of object recognition tasks. In summary, although females are commonly described with deficits and the articles emphasize sex differences, most published data reveal similar performances when sexes are compared.

## Introduction

Historically, female subjects are neglected in biomedical science. Particularly, in neuroscience, over 5 males are used for each female, and the reason to avoid females is the alleged variation due to their reproductive cycles (Zucker and Beery, 2010). However, sexual features are relevant biological variables (National Institute of Health [NIH], 2015), and the inclusion of equal numbers of both the sexes in the studies is recommended. Female and male animals can exhibit completely different responses in the same behavioral task (Ribeiro et al., 2010). Therefore, we should not only include females, but be aware of the peculiarities of this sex. Specifically, there is a common sense that females do not perform as well as males in memory tasks (particularly in spatial memory) (Vorhees and Williams, 2014). In addition, most of the studies use procedures to control or suppress the natural female hormone cycle, such as vaginal lavage procedure (VLP) or ovariectomy, regardless of the consequences of these manipulations, which are insufficiently studied. Even considering evidence that female’s performance is worse, some of the reasons that could explain this fact beyond a cognitive difference *per se* are: (1) most, if not all, tasks are standardized for males; (2) the manipulations performed only in females could result in misinterpretation of data, if not controlled; and (3) publication bias, as both the authors and journals show a preference for publication of positive over negative results (sex differences over sex similarities). Thus, the equalization of the number of subjects between sexes is not enough. More attention should be paid to methods of including females, adequate controls, and interpretation of results without considering male’s performance the “normal” one. Finally, it is important to consider comprehensive surveys of the literature when discussing sex comparisons or female behavior.

Four versions of object recognition’s task are used in the studies selected for the present review: (1) Novel object recognition (NOR): rats are presented to 2 identical objects in the training session, and in the test session one object is changed for a new object; it is expected that the rat explores more the novelty (Abbott et al., 2016); (2) Place recognition: rats are presented to 2 identical objects in the training session, and in the test session one object is in a different position, which adds a spatial aspect to the task; it is expected that the rat explores more the moved object (Abbott et al., 2016); (3) Object-in-place recognition (OIPR): there are 4 different objects in the training session, and in the test session 2 of those objects exchange places; this version combines the spatial aspect with the object identification; it is expected that the rat explores more the reallocated objects (Abbott et al., 2016); and (4) Object-in-context recognition (OICR): rats are presented to 2 identical objects in a context A (for example, dark room and dark apparatus), then presented to 2 new identical objects in context B (for example, bright room, and bright apparatus); afterward, rats are placed in context A or B with 1 object of each context; it is expected that the rat explores more the object presented in a context different from the one it was first seen (Lee et al., 2014). There are other versions of object recognition tasks that have not been explored in female animals yet. For example, some protocols consider the order of objects presented as a temporal aspect of recognition memory (Barbosa et al., 2012).

It is known that sex and sex steroids impact recognition tasks, and that females’ performance can differ from males in NOR tasks (McCarthy et al., 2018). Recognition tasks can be used in the study of diseases such as brain injuries, attention-deficit hyperactivity disorder, or Alzheimer’s disease (which differs between sexes in several aspects—de Macêdo Medeiros and Silva, 2019). Moreover, these tasks are also relevant for studying functional neuroanatomy, aging, and the role of neurotransmitters, which reinforce the need for studying both the sexes (Ennaceur and Silva, 2018).

Our study aimed to review published articles that used object recognition tasks to verify sex similarities and differences. Besides the reduced number of studies that include females, we discuss possible constraints of the studies that can be crucial to the interpretation of females’ behavior, such as manipulations that are exclusive to this sex. Therefore, we expect to incentivize the inclusion of both the sexes in object recognition studies, with adequate approaches to study female rats’ behavior and compare performances between sexes.

## Methods

The studies were selected using the PubMed database (accessed on 12 October 2021).^1^ The search terms were “sex differences and object recognition and rat” and the filter for “other animals” was used. Articles that did not use rats, did not test males and females in the same task, considered the data of males and females together for analysis, were not clear about the sex of animals used, did not include an object recognition task, or did not include the control groups with no previous manipulation not related to the estrous cycle were excluded from the survey. Every article comparing male and female rats in a version of object recognition task with groups that had no previous manipulation was included.

## Results

A preliminary search returned 6,662 articles when the term “object recognition” was combined with the PubMed filter “other animals.” When we added the filter “females,” 1,567 articles were listed, suggesting 23.52% of the articles in the first search included females. This percentage of studies, including females, may not look much, but in the field of neuroscience, the proportion is usually five males for each female (Zucker and Beery, 2010), revealing that articles on object recognition tasks are not particularly sex-biased.

**FIGURE 1:**
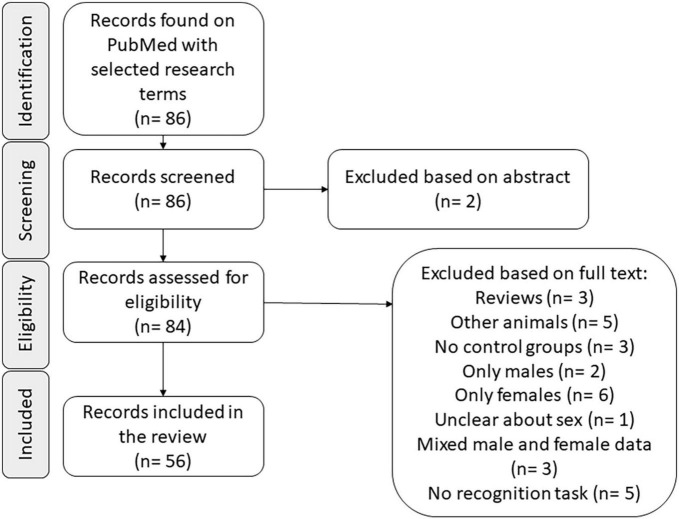
Graphical flow diagram of the article selection process (n: number of articles in each phase).

The main search was conducted according to the detailed criteria described above, and 56 articles were selected (see Figure 1 and Table 1). Most of the selected articles included groups submitted to manipulations not related to sex; those groups were not considered in our analysis.

**TABLE 1:** Summarized information of the selected articles.

References	Title	Strain (age during the test)	Manipulations that could have impacted sex comparisons	Recognition task	Outcome of control animals
Abbott et al., 2016	Sex-specific effects of daily exposure to sucrose on spatial memory performance in male and female rats, and implications for estrous cycle stage	Sprague Dawley (3 months old)	VLP	Novel object	Both male and female differentiated the objects
				Place	Both male and female differentiated the objects
				Object-in-place	Both male and female differentiated the objects
Arfa-Fatollahkhani et al., 2017	The effect of luteinizing hormone reducing agent on anxiety and novel object recognition memory in gonadectomized rats	Wistar (4 months old)	OVX + HR and unclear about VLP	Novel object	Both male and female differentiated the objects
Alteba et al., 2016	Cannabinoids reverse the effects of Early stress on neurocognitive performance in adulthood	Unclear (20 days old)	Unclear	Novel object	Both male and female differentiated the objects
				Place	Both male and female differentiated the objects
Anselmi et al., 2016	Genetic evidence for chromosome 4 loci influencing learning and memory	Sprague Dawley, LEW and SHR (after 11 weeks old)	Unclear	Novel object	Both male and female differentiated the objects
Braun et al., 2018	Sex-specific effects of Cacna1c haploinsufficiency on object recognition, spatial memory, and reversal learning capabilities in rats	Sprague Dawley (94 day old)	Unclear	Novel object	Both male and female differentiated the objects
Barbie-Shoshani et al., 2016	Sex-specific effects of prenatal stress on memory and markers of neuronal activity in juvenile rat	Wistar (24–32 days old)	Unclear	Novel object	Both male and female differentiated the objects
Bengoetxea et al., 2017	Effects of perinatal diet and prenatal stress on the behavioral profile of aged male and female rats	Wistar (1 and 19 months old)	Unclear	Novel object	Both male and female differentiated the objects
Beck and Luine, 2002	Sex differences in behavioral and neurochemical profiles after chronic stress: Role of housing conditions	Sprague Dawley (50–60 days old)	Unclear	Novel object	Females learned regardless of housing
				Place	Only males learned
Bowman et al., 2006	Aged rats: Sex differences and responses to chronic stress	Sprague Dawley (20 months old)	Unclear	Novel object	Both male and female differentiated the objects
				Place	Nor male or female differentiated the objects, not included
Baran et al., 2010	Prefrontal cortex lesions and sex differences in fear extinction and perseveration	Sprague Dawley (age unspecified, weight 275–300 g)	VLP	Novel object	Males retained the memory for longer
				Place	Both male and female differentiated the objects
Bowman et al., 2009	Sex-dependent changes in anxiety, memory, and monoamines following 1 week of stress	Sprague Dawley (8 weeks old)	Unclear	Novel object	Both male and female differentiated the objects
				Place	Only males learned
Bruijnzeel et al., 2019	Effects in rats of adolescent exposure to cannabis smoke or THC on emotional behavior and cognitive function in adulthood	Long Evans (129 days old)	Unclear	Novel object	Both male and female differentiated the objects
Bowman et al., 2015	Bisphenol-A exposure during adolescence leads to enduring alterations in cognition and dendritic spine density in adult male and female rats	Sprague Dawley (5 weeks old)	Unclear	Novel object	Both male and female differentiated the objects
				Place	Both male and female differentiated the objects
Cost et al., 2012	Sex differences in object-in-place memory of adult rats	Long evans (55–60 days old)	VLP or OVX + HR	Object-in-place	Females submitted to VLP didn’t learn
Cyrenne and Brown, 2011a	Ontogeny of sex differences in response to novel objects from adolescence to adulthood in lister-hooded rats	Lister hooded (28–80 days old)	Unclear	Novel object	Males learned in every age tested, females didn’t learn by the age of 40 day
Cyrenne and Brown, 2011b	Effects of suppressing gonadal hormones on response to novel objects in adolescent rats	Lister hooded (40 days old)	Unclear	Novel object	Both male and female differentiated the objects, males had higher preference
Ellis et al., 2020	Paternal morphine self-administration produces object recognition memory deficits in female, but not male offspring.	Sprague Dawley (age unspecified, weight 250–300 g)	Unclear	Novel object	Both male and female differentiated the objects
				Place	Females learned, males were not tested, not included
Ennaceur et al., 2005	Detailed analysis of the behavior of lister and Wistar rats in anxiety, object recognition and object location tasks	Long evans and Wistar (2 months old)	Unclear	Novel object	Both male and female differentiated the objects
				Place	Both male and female differentiated the objects
Fielding et al., 2012	Profiles of motor and cognitive impairment in the transgenic rat model of Huntington’s disease	Sprague Dawley (22 months)	Unclear	Novel object	Both male and female differentiated the objects
Foley et al., 2014	Sexually dimorphic effects of prenatal exposure to propionic acid and lipopolysaccharide on social behavior in neonatal, adolescent, and adult rats: Implications for autism spectrum disorders	Long evans (43 days old)	Unclear	Novel object	Both male and female differentiated the objects, females made more visits to the objects, and spent more time with the objects
Gillera et al., 2021	Sex-specific effects of Perinatal FireMaster^®^ 550 (FM 550) exposure on socioemotional behavior in prairie voles	Unclear (80 days old)	Unclear	Novel object	Both male and female differentiated the objects
Ghi et al., 1999	Sex differences in memory performance in the object recognition test. Possible role of histamine receptors	Wistar (40 days old)	Unclear	Novel object	Females retained the memory for longer
Gonzales et al., 2015	Repeated neonatal propofol administration induces sex-dependent long-term impairments on spatial and recognition memory in rats.	Kyoto Wistar (6 weeks old)	Unclear	Novel object	Both male and female differentiated the objects
Howland et al., 2012	Altered object-in-place recognition memory, prepulse inhibition, and locomotor activity in the offspring of rats exposed to a viral mimetic during pregnancy	Long evans (60–90 days old)	VLP	Novel object	Both male and female differentiated the objects
				Place	Both male and female differentiated the objects, males had higher preference
				Object-in-place	Both male and female differentiated the objects
Hill et al., 2014	Sex-specific disruptions in spatial memory and anhedonia in a “two hit” rat model correspond with alterations in hippocampal brain-derived neurotrophic factor expression and signaling	Wistar (6 weeks old)	Unclear	Novel object	Both male and female differentiated the objects
Jordan and Andersen, 2018	Working memory and salivary brain-derived neurotrophic factor as developmental predictors of cocaine seeking in male and female rats	Sprague Dawley (20 days)	None	Novel object	Both male and female differentiated the objects
Klambatsen et al., 2019	Sex differences in memory and intracellular signaling after methamphetamine binge treatment	Sprague Dawley (8 weeks old)	OVX and unclear about VLP	Novel object	Both male and female differentiated the objects
Kolyaduke and Hughes, 2013	Increased anxiety-related behavior in male and female adult rats following early and late adolescent exposure to 3,4-methylenedioxymethamphetamine (MDMA)	PVG/C hooded (90 days old)	Unclear	Novel object	Both male and female differentiated the objects
Klug and van den Buuse, 2012	Chronic cannabinoid treatment during young adulthood induces sex-specific behavioral deficits in maternally separated rats	Wistar (8 weeks old)	Unclear	Novel object	Both male and female differentiated the objects
Lian et al., 2018	Object, spatial and social recognition testing in a single test paradigm.	Sprague Dawley (age unspecified, weight 170–200 g)	Unclear	Novel object	Both male and female differentiated the objects
				Place	Both male and female differentiated the objects
Macht et al., 2020	Adolescent alcohol exposure produces protracted cognitive-behavioral impairments in adult male and female rats	Long evans (9 months old)	Unclear	Novel object	Both male and female differentiated the objects
Mansouri et al., 2012	Gender-dependent behavioral impairment and brain metabolites in young adult rats after short term exposure to lead acetate	Wistar (55–60 days old)	Unclear	Novel object	Both male and female differentiated the objects
Marco et al., 2013	Maternal deprivation effects on brain plasticity and recognition memory in adolescent male and female rats	Wistar (after 22 days old)	Unclear	Novel object	Both male and female differentiated the objects
Muhammad and Kolb, 2011	Mild prenatal stress-modulated behavior and neuronal spine density without affecting amphetamine sensitization	Long evans (30–40 days old)	Unclear	Novel object	No effect of sex
Muhammad et al., 2011	Tactile stimulation during development attenuates amphetamine sensitization and structurally reorganizes prefrontal cortex and striatum in a sex-dependent manner	Long evans (30–40 days old)	Unclear	Novel object	Both male and female differentiated the objects
Mourlon et al., 2010	Maternal deprivation induces depressive-like behaviors only in female rats	Long evans (68–111 days old)	Unclear	Novel object	Both male and female differentiated the objects, female would learn exploring less the objects during the training phase
Nelson et al., 2018	Chronic moderate alcohol drinking alters insulin release without affecting cognitive and emotion-like behaviors in rats	Long evans (23 days old)	Unclear	Novel object	Both male and female differentiated the objects
Pereira et al., 2008	Early enriched housing results in partial recovery of memory deficits in female, but not in male, rats after neonatal hypoxia-ischemia	Wistar (30 days old)	Unclear	Novel object	Both male and female differentiated the objects
Paris and Frye, 2011	Juvenile offspring of rats exposed to restraint stress in late gestation have impaired cognitive performance and dysregulated progestogen formation	Long evans (28–30 days old)	Unclear	Novel object	Both male and female differentiated the objects
Peay et al., 2021	Chronic unpredictable intermittent restraint stress disrupts spatial memory in male, but not female rats	Sprague Dawley (age unspecified, weight 200–225 g)	Unclear	Novel object	Both male and female differentiated the objects
				Place	Both male and female differentiated the objects
Reichel et al., 2012	Sex differences in escalation of methamphetamine self-administration: Cognitive and motivational consequences in rats	Long evans (age unspecified, males’ weight 250–300 g, females’ 180–200 g)	Unclear	Novel object	Both male and female differentiated the objects
				Object-in-place	Both male and female differentiated the objects
Robison et al., 2017	Sex differences in the physiological and behavioral effects of chronic oral methylphenidate treatment in rats	Sprague Dawley (4 weeks old)	Unclear	Novel object	Both male and female differentiated the objects, female would learn exploring less the objects during the training phase
Saucier et al., 2008	Sex differences in object location memory and spatial navigation in long-evans rats.	Long evans hooded (50 days old	Unclear	Object-in-place	Both male and female differentiated the objects, female would learn exploring less the objects during the training phase
Salomon et al., 2011	Corticosterone mediates some but not other behavioral changes induced by prenatal stress in rats	Wistar (31 days old)	VLP	Novel object	Only females learned
Salas-Ramirez et al., 2010	Prenatal cocaine exposure increases anxiety, impairs cognitive function and increases dendritic spine density in adult rats: influence of sex	Sprague Dawley (64–68 days old)	VLP	Novel object	Both male and female differentiated the objects
				Place	Both male and female differentiated the objects
Santollo et al., 2019	Gonadal hormones in female rats protect against dehydration-induced memory impairments in the novel object recognition paradigm	Sprague Dawley (age unspecified, weight 75–100 g)	VLP or OVX	Novel object	Both male and female differentiated the objects
Sallaberry et al., 2018	Sex differences in the effects of pre- and post-natal caffeine exposure on behavior and synaptic proteins in pubescent rats	Wistar (35 and 70 days old)	Unclear	Novel object	Only females learned
Sadegzadeh et al., 2020	Effects of adolescent administration of fluoxetine on novel object recognition memory, anxiety-like behaviors, and hippocampal brain-derived neurotrophic factor level	Wistar (2–3 months old)	Unclear	Novel object	Both male and female differentiated the objects
Sutcliffe et al., 2007	Influence of gender on working and spatial memory in the novel object recognition task in the rat	Hooded lister (age unspecified, weight 234–373 g)	VLP	Novel object	Females retained the memory for longer
				Place	Estrous cycle’s phases interfered in female behavior
Turgeon et al., 2016	Chronic caffeine produces sexually dimorphic effects on amphetamine-induced behavior, anxiety and depressive-like behavior in adolescent rats	Sprague Dawley (44 days old)	Unclear	Novel object	Both male and female differentiated the objects
van Goethem et al., 2012	Object recognition testing: Rodent species, strains, housing conditions, and estrous cycle	Wistar (4 months old)	VLP	Novel object	Both male and female differentiated the objects
Villanueva Espino et al., 2020	Cognitive training increases dendritic arborization in the dorsal hippocampal CA1 and CA3 neurons of female and male Long–Evans rats	Long evans (56 days old)	Unclear	Novel object	Both male and female differentiated the objects
Weston et al., 2014	Sex-dependent and Non-monotonic enhancement and unmasking of methylmercury neurotoxicity by prenatal stress	Long evans (3 months old)	Unclear	Novel object	Both male and female differentiated the objects
Wooden et al., 2021	A sensitive homecage-based novel object recognition task for rodents	Long Evans (70 days old)	Unclear	Novel object	Both male and female differentiated the objects, female would learn exploring less the objects during the training phase
Winther et al., 2018	Maternal high-fat diet programs offspring emotional behavior in adulthood	Sprague Dawley (7 weeks old)	Unclear	Novel object	Both male and female differentiated the objects
Zamberletti et al., 2012	Gender-dependent behavioral and biochemical effects of adolescent delta-9-tetrahydrocannabinol in adult maternally deprived rats	Sprague Dawley (65 days old)	Unclear	Novel object	Both male and female differentiated the objects

VLP, vaginal lavage procedure; OVX, ovariectomy; HR, hormonal reposition.

### Novel object recognition

Most articles revealed that female and male had similar performances considering discrimination ratio (Ennaceur et al., 2005; Salas-Ramirez et al., 2010; Muhammad et al., 2011; Muhammad and Kolb, 2011; Fielding et al., 2012; Howland et al., 2012; Klug and van den Buuse, 2012; Mansouri et al., 2012; van Goethem et al., 2012; Zamberletti et al., 2012; Kolyaduke and Hughes, 2013; Marco et al., 2013; Hill et al., 2014; Abbott et al., 2016; Alteba et al., 2016; Anselmi et al., 2016; Barbie-Shoshani et al., 2016; Turgeon et al., 2016; Arfa-Fatollahkhani et al., 2017; Bengoetxea et al., 2017; Jordan and Andersen, 2018; Lian et al., 2018; Winther et al., 2018; Bruijnzeel et al., 2019; Klambatsen et al., 2019; Sadegzadeh et al., 2020), percentage of time—relative time exploring the novel object considering total amount of object exploration (Pereira et al., 2008; Bowman et al., 2009, 2015; van Goethem et al., 2012; Nelson et al., 2018; Ellis et al., 2020; Macht et al., 2020; Gillera et al., 2021; Peay et al., 2021), or absolute time—duration of novel object exploration (Paris and Frye, 2011; Reichel et al., 2012; Weston et al., 2014; Gonzales et al., 2015; Braun et al., 2018; Santollo et al., 2019; Villanueva Espino et al., 2020).

Some studies demonstrated that females were better than males, based on one of these outcomes: only females preferred the novelty (Salomon et al., 2011; Sallaberry et al., 2018), females learned regardless of housing, while only single-housed males (Beck and Luine, 2002), females learned even if exploring less the objects during training (Mourlon et al., 2010; Robison et al., 2017; Wooden et al., 2021), females made more visits and spent more time exploring the novelty (Foley et al., 2014), or females retained the memory for a longer period (Ghi et al., 1999; Sutcliffe et al., 2007). On the other hand, some studies showed that males were better than females due to the following results: males retained the memory for a longer period (Baran et al., 2010), only males learned when aged 40 days, while both the sexes learned at other ages (Cyrenne and Brown, 2011a DevP), or males had higher discrimination indexes (Cyrenne and Brown, 2011b).

### Place recognition

Most articles revealed that females and males had similar performances considering the discrimination ratio (Ennaceur et al., 2005; Baran et al., 2010; Salas-Ramirez et al., 2010; Abbott et al., 2016; Alteba et al., 2016) and the percentage of time exploring objects (Bowman et al., 2015; Peay et al., 2021). Some studies concluded that males were better than females because females did not differentiate the objects (Beck and Luine, 2002; Bowman et al., 2009), only estrus females learned (Sutcliffe et al., 2007), or males had higher discrimination indexes (Howland et al., 2012).

### Object-in-place recognition

Most articles revealed that females and males had similar performances considering the discrimination ratio (Howland et al., 2012; Abbott et al., 2016) and absolute time (Reichel et al., 2012). One of the studies demonstrated that females did not discriminate the objects when submitted to VLP, but learns when submitted to ovariectomy, while intact and unstressed males learned the task (Cost et al., 2012). Another study demonstrated that male and female rats differentiated the objects, despite females exploring less the objects during training (Saucier et al., 2008).

### Object-in-context recognition

Although we did not find studies comparing male and female animals in OIPR tasks, an article using only females revealed that control rats learn this task considering discrimination index and time exploring the objects (Sasaki Russell et al., 2019).

## Discussion

Most published articles revealed similar performances, but some articles suggested that females performed NOR better than males, and males performed PR better than females. Although this evidence is not robust considering all the studies together, these findings corroborate human studies in which females are better in object or color recognition and males are better in location recognition (McGivern et al., 2019). Regarding OIPR, literature does not show any sex as having better performance (see Table 2).

**TABLE 2:** Number of articles revealing no differences, benefiting males, and benefiting females.

	Frequency	Percentage
**Novelty object recognition**		
No differences	41	78%
Benefits males	3	6%
Benefits females	9	16%
Total	54	100%
**Place recognition**		
No differences	9	69%
Benefits males	4	31%
Benefits females	0	0%
Total	13	100%
**Object-in-place recognition**		
No differences	3	60%
Benefits males	1	20%
Benefits females	1	20%
Total	5	100%

For all the three tasks, most published articles did not show any differences between female and male behaviors.

Some methodological aspects can hinder the collective interpretation of the selected articles: (1) the use of the discrimination index (also referred to as ratio). This parameter is commonly understood as (time exploring new object–time exploring old object)/(total exploration time), but many studies claim to use a discrimination index, but actually reported the percentage of time exploring the object, which can be confusing and makes it harder to compare the data; (2) many articles were not clear about the age of animals, which can lead to variability in the behavior; (3) the interpretation of the researchers is likely to consider non-significant data or tendencies that benefit males such as the time spent near the objects (Ceccarelli et al., 2001) or emphasizing females exhibited a lower discrimination index when they learn the task (Cyrenne and Brown, 2011b) or when neither male nor female spent more time exploring the new object (Bowman et al., 2006); and (4) even when there is a significant difference, most articles do not include the effect size in order to highlight the relevance of the behavioral difference.

Importantly, the absence of control groups in many published articles is a relevant issue, especially considering females’ performances. Similar to what is done for male animals, the female control group must be free of specific stressors and manipulations, i.e., studies should include a group of female rats that are not submitted to VLP or ovariectomy/hormonal reposition. Indeed, many animals had been previously submitted to these manipulations, without including female rats that did not go through those procedures. In addition, most articles did not make it clear if they used these manipulations or how they evaluated the consequences of that use (Klambatsen et al., 2019; Santollo et al., 2019). Some of them used females submitted to VLP as controls and compared them to intact males and gonadectomized females (Cost et al., 2012). In this respect, it has been shown that estrous cycle monitoring is stressful (Becegato et al., 2021) and alters female behavior (Walker et al., 2002; Becegato et al., 2021). Only one of the selected articles highlighted that they avoided VLP because of the possibility of altering behavior (Jordan and Andersen, 2018). Another study using mice assessed the estrous cycle using the visual method daily and performed a single VLP to confirm the phase (Mitra et al., 2017), as proposed by Walker et al. (2002). Thus, few researchers that studied object recognition have shown adequate approaches to deal with particularities of studying behavior in females. Many studies compare stressed females (caused by VLP) to unstressed males, whereas it is well known that stress has a major impact on spontaneous behavior (Klenerová et al., 2007; Rabelo-da-Ponte et al., 2019) and memory (for a review, see Cazakoff et al., 2010). Thus, this is a major weakness of these studies. Hence, we suggest the addition of a control group of naïve females, which are not submitted to any manipulations regarding their hormonal fluctuations; in the same way, intact males are usually included as controls.

Two of the articles selected have included both the females that were monitored with VLP and ovariectomized females. In Cost et al.’s (2012) article, those female groups were compared to intact males in the OIPR task. The results showed that females that were submitted to VLP and tested in the diestrus phase had worse performance (decreased delay of retention) compared to males, while vehicle-treated ovariectomized females had similar performance compared to males. In Santollo et al.’s (2019) study, cycling females were compared to intact males and gonadectomized females in the NOR task. In one of the experiments, VLP females tested in the diestrus or estrus cycle presented performance comparable to males. In another experiment, the behavior of intact and ovariectomized females was similar, but it is not clear if intact females were submitted to estrous cycle monitoring. In another study, Klambatsen et al. (2019) compared ovariectomized females and intact females in the NOR task; both the groups differentiated the objects and spent a larger percentage of time with the novel object, but it is not clear if intact females were submitted to estrous cycle monitoring (Klambatsen et al., 2019).

A few articles evaluated the possible influence of the estrous cycle’s phases on the performance of female rats. It has been shown that metestrus and diestrus females learned PR and OIPR tasks (Abbott et al., 2016). However, it has also been shown that only estrus rats preferred the moving object in the PR task (Sutcliffe et al., 2007) and that diestrus females only retained the memory of OIPR for 5 min (Cost et al., 2012). Regarding the NOR task, rats in all the phases showed adequate performance (Sutcliffe et al., 2007; van Goethem et al., 2012), but metestrus and diestrus animals had smaller discrimination indexes compared to proestrus and estrus animals (van Goethem et al., 2012). Thus, the differences and similarities in females’ behavior across the estrous cycle are still unclear. Importantly, as mentioned, monitoring the estrous cycle involves a stressful procedure that could interact with the hormonal status to influence behavior. Overall, most of the articles were not clear about the evaluation of the estrous cycle’s phases, and the ones that presented those data were far from unanimous. Importantly, the manipulations used to evaluate the estrous cycle phase can alter rat’s behavior and even mask existing differences between the phases (Walker et al., 2002). On the other hand, ovariectomy does not seem to impair NOR and OIPR tasks (Cost et al., 2012; Arfa-Fatollahkhani et al., 2017; Klambatsen et al., 2019).

It is relevant to highlight that most published articles did not describe the details of ovariectomy or VLP, which makes reproducibility difficult. For example, some articles did not inform the method chosen for estrous cycle monitoring (Salas-Ramirez et al., 2010; Salomon et al., 2011; Santollo et al., 2019). Frequently, it was not clear how many times VLP was performed (Baran et al., 2010; van Goethem et al., 2012), and post-surgical care was not always well described (Arfa-Fatollahkhani et al., 2017). In addition, sometimes ovariectomy surgery is barely cited (Klambatsen et al., 2019). Few articles had a simple but reasonable explanation for their methods choice (Cost et al., 2012; Abbott et al., 2016). These methodological description constraints involving VLP and ovariectomy can lead to difficulties in the interpretation of the studies, and the differences or similarities described in Table 2 might be unrealistic.

It is relevant to point out the relevance of the terms sex and gender when performing literature surveys. Gender refers to the social roles, socialization, and expressions, and, hence, applicable only to human studies. In animal studies, sex should be used, as it refers to biological aspects such as chromosomes, genes, hormones, gonads, and genitals. Nevertheless, as this conceptualization is somewhat recent, some published articles use “gender” when referring to animals (Sutcliffe et al., 2007).

Finally, there are recent articles that still are not clear about the sex of the animals used or mix male and female data without a reasonable justification. An adequate form of mixing data from both the sexes is the work by Arbogast et al. (2019). They planned a cohort with male and female animals in a 50:50 sex ratio; then, they first evaluated the performances separately. Since no significant sex differences were found, they mixed the data of both the sexes. Authors should provide accurate descriptions of all aspects of the methods used in the studies.

In conclusion, the present literature review raises several aspects of object recognition studies with female subjects that can lead to flawed interpretations, such as the consideration of non-significant data that benefit males, the absence of appropriate control groups, and the use of manipulations that interfere with female physiology and behavior without considering these effects. However, even with those confounding factors, most data show that females learn all the types of recognition tasks and most data reveal no sex differences in the performance of these tasks. This outcome not only highlights the importance of including females in behavioral studies, but also indicates that comprehensive reviews can be important tools to discuss and interpret sex differences in neuroscience.

## Author contributions

MB collected the data, performed the analysis, and wrote the study. RS coordinated the study and revised the manuscript. Both authors contributed to the article and approved the submitted version.

## Footnotes

1
https://pubmed.ncbi.nlm.nih.gov/
